# First Report of Integrative Conjugative Elements in *Riemerella anatipestifer* Isolates From Ducks in China

**DOI:** 10.3389/fvets.2019.00128

**Published:** 2019-04-24

**Authors:** Dekang Zhu, Jianbang Wan, Zhishuang Yang, Jinge Xu, Mingshu Wang, Renyong Jia, Shun Chen, Mafeng Liu, Xinxin Zhao, Qiao Yang, Ying Wu, Shaqiu Zhang, Yunya Liu, Ling Zhang, Yanling Yu, Xiaoyue Chen, Anchun Cheng

**Affiliations:** ^1^Research Center of Avian Diseases, College of Veterinary Medicine, Sichuan Agricultural University, Chengdu, China; ^2^Key Laboratory of Animal Disease and Human Health of Sichuan Province, Chengdu, China; ^3^Institute of Preventive Veterinary Medicine, Sichuan Agricultural University, Chengdu, China; ^4^Guizhou Animal Husbandry and Veterinary Research Institute, Guiyang, China

**Keywords:** *Riemerella anatipestifer*, integrative conjugative elements, cargo genes, antimicrobial resistance, virulence

## Abstract

We report for the first time the occurrence of integrative conjugative elements (ICEs) in *Riemerella anatipestifer* (*R.anatipestifer*) isolated from diseased ducks in China. For this purpose, a total of 48 genome sequences were investigated, which comprised 30 publicly available *R. anatipestifer* genome sequences, and 18 clinical isolates genomes sequences. Two ICEs, named ICE*Ran*RCAD0133-1 and ICE*Ran*RCAD0179-1 following the classic nomenclature system, were identified in *R. anatipestifer* through the use of bioinformatics tools. Comparative analysis revealed that three ICEs in *Ornithobacterium rhinotracheale* showed a high degree of conservation with the core genes of ICE*Ran*RCAD0133-1, while 13 ICEs with high similarity to ICE*Ran*RCAD0179-1 were found in *Bacteroidetes*. Based on the definition of ICE family, ICE*Ran*RCAD0179-1 was grouped in CTn*DOT/ERL* family; however, ICE*Ran*RCAD0133-1, which had no significant similarity with known ICEs, might be classified into a novel ICE family. The sequences of ICE*Ran*RCAD0133-1 and ICE*Ran*RCAD0179-1 were 70890 bp and 49166 bp in length, had 33.14 and 50.34% GC content, and contained 77 CDSs and 51 CDSs, respectively. Cargo genes carried by these two ICEs were predicted to encode: R-M systems, IS elements, a putative TonB-dependent receptor, a bacteriocin/lantibiotic efflux ABC transporter, a tetracycline resistance gene and more. In addition, phylogenetic analyses revealed that ICE*Ran*RCAD0179-1 and related ICEs were derived from a common ancestor, which may have undergone divergence prior to integartation into the host bacterial chromosome, and that the core genes co-evolved via a related evolutionary process or experienced only a low degree of recombination events during spread from a common CTn*DOT*/*ERL* family ancestor. Collectively, this study is the first identification and characterization of ICEs in *R. anatipestifer*; and provides new insights into the genetic diversity, evolution, adaptation, antimicrobial resistance, and virulence of *R. anatipestifer*.

## Introduction

Integrative conjugative elements (ICEs), a type of self-transmissible mobile genetic elements (MGEs) are widely distributed in bacterial genomes. ICEs are major mediators for horizontal gene transfer (HGT), which contribute to microbial evolution and adjustment to new niche (1, 2). ICEs have the capability to excise from their host chromosome and reintegrate into a new host's chromosome at a target site where they replicate as a part of the host chromosome. Generally, each type of ICE contains a set of core genes involved in its integration and excision, regulation and maintenance, and conjugative transfer (3). The remaining components of ICEs are accessory genes, which endow the host bacteria with multiple phenotypes that can be beneficial for the recipient bacteria, such as colonization of a eukaryotic host, nitrogen fixation or promotion of virulence and biofilm formation, or resistance to antibiotics and heavy metals (4). In particularly, homologous recombination among unrelated or distantly related ICEs probably occurs in ICE recipients, resulting in a diverse set of novel hybrid ICEs, which leads to the diversity of ICEs among bacterial genomes (5, 6).

*R.anatipestifer*, a member of the *Flavobacteriaceae* family, is a non-spore-forming and rod-shaped Gram-negative bacterium. It is a major contributor to acute and chronic septicemia associated with fibrinous pericarditis, perihepatitis, airsacculitis, and meningitis (7). *R.anatipestifer* can infect various types of avian species, such as ducks, geese, turkeys, and some wild birds. *R.anatipestifer* infection is a contagious disease, that causes enormous economic losses to the poultry industry worldwide (8).

Here, we investigated 48 *R.anatipestifer* genome sequences by using bioinformatics tools, comprised of 30 publicly available genomic sequences and 18 clinical isolates genomes sequenced in this study. As a result, two ICEs were identified in the genome of *R.anatipestifer* (RCAD0133 and RCAD0179) for the first time. Analysis of coding sequences (CDS) within the accessory regions of the two ICEs identified several putative virulence factors and an antibiotic resistance gene, which can confer hosts with selective advantages beneficial for niche adaptation. Alignment and comparative analysis of those two ICEs revealed that homologous ICE-like elements are prevalent in other bacterial genomes, and shed light on their phylogenetic relationships and on their modular evolution.

## Methods and Materials

### Bacterial Isolates and DNA Purification

A total of 18 clinical isolates of *R.anatipestifer* were obtained from the livers of infected ducks and identified using PCR. Among them, 9 strains obtained from Culture Collection of the University of Gothenburg (CCUG) (Table S1). All animal studies were conducted in accordance with the Guide for the Care and Use of Laboratory Animals. The duck-use procedures were approved by the Animal Ethics Committee of the Sichuan Agricultural University (approval No. 2017-013). All isolates were purely cultured for genome sequencing. Genomic DNA was extracted using a TIANamp Bacteria DNA Kit (Tiangen Biotech Co, Ltd., Beijing, China), which was further quantified using a NanoDrop 2000 (Thermo-Scientific).

### Genome Sequencing, Assembly, and Annotation

The sequence of strain RCAD0133 was determined by applying a single-molecule real-time sequencing platform with a PacBio RS II sequencer. The raw data were filtered, and then, a *de novo* assembly was executed with MHAP (9). The remaining 17 strains were sequenced using an Illumina HiSeq 2500 instrument. A *de novo* assembly was conducted using Velvet version 1.2.09 (10). The 18 assembled sequences were annotated by the NCBI Prokaryotic Genome Annotation Pipeline (PGAP) (http://www.ncbi.nlm.nih.gov/genome/annotation_prok). The average nucleotide identity (ANI) of all the R. anatipestifer involved in this study was calculated by pyani (a Python module, https://github.com/widdowquinn/pyani) with the ANIb algorithm.

### *In silico* Characterization of the ICE Elements in *R.anatipestifer*

All 48 *R.anatipestifer* genome sequences (18 were newly sequenced and 30 sequences were publicly available in the NCBI database) were analyzed using bioinformatics tools. Islandviewer 4 was used to predict genomic islands; also the secretion systems were identified via MacSyFinder, then the genomic islands containing a type IV secretion system (T4SS) (Table S2) and a relaxase were extracted as inputs for the ICEberg server to accurately confirm potential ICEs, and identify which ICE family they belong to (11–14). ICEs integration sites were determined by searching direct repeats (DRs) using a BLASTn alignment of the first and last 400 bp of the predicted ICEs. The ORF sets encoded on the ICE*Ran* elements were standardized using the PATRIC annotation tool (15, 16). BLASTp was used to determine the putative functions of the hypothetical proteins encoded by ORFs on ICE elements against the nr database, while conserved domains were identified using the Batch CD-search tool and the Conserved Domain Database (17, 18). Easyfig was used to visualize ICEs organization and their comparisons (19). For amino acid alignments, putative proteins with hits below 40% identity or alignment <120 base pairs were discarded (20).

### Search for the Homologs of ICE*Ran*RCAD0133-1 and ICE*Ran*RCAD0179-1 in Related Strains

Searching homologous regions (ICE-like elements) for the putative ICE were performed by NCBI BLASTn comparison analysis using the original confirmed ICE sequence as a probe against the NCBI non-redundant nucleotide sequences(nr) database and the whole-genome shotgun contigs (wgs) database limited by *Riemerella* taxon.

### Phylogenetic Tree Construction

The amino acid sequences of the ICE host Chromosomal housekeeping genes *infB, gyrB*, and *rpoB*; ICE elements core genes and the analogous ICE-like elements core genes were extracted, concatenated, and aligned using MUSCLE (21). A phylogenetic tree was constructed using MEGA v 7.0 with the neighbor-joining method, by Poisson correction and a bootstrap test was performed with 1,000 replications (22).

### Nucleotide Sequence Accession Number

All 18 newly sequenced nucleotide sequences of *R.anatipestifer* in this work were submitted to GenBank and all the accession numbers of investigated *R.anatipestifer* sequences are listed in the supplementary file (Table S1). For the ANI analysis of 48 *R.anatipestifer*, the minimum ANI was 93% (Supplementary Figure 1).

## Results

### General Properties of Integrative Conjugative Elements in *R.anatipestifer*

**Figure 1:**
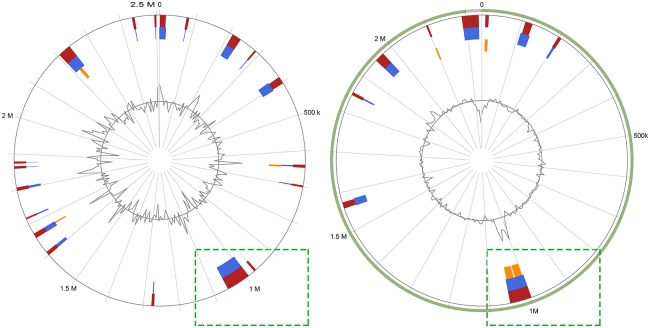
Identification of genomic islands (candidate integrative conjugative elements) Islandviewer 4 analyses of the *R.anatipestifer* RCAD0133 and *R. anatipestifer* RCAD0179 chromosomes, with colored regions indicating genomic islands which likely are integrative conjugative elements.

All 18 novel genome sequences obtained in this study and other publicly available genome sequences from the NCBI database (Table S1) were submitted to the Islandviewer4 server. Among these predicted genomic islands (Table S3, Table S4), two speculative integrative conjugative elements were then confirmed by the ICEberg server (Figure 1) (11, 14). Interestingly, an incomplete ICE was split into two contigs within the RA-JLLY genome sequence, which may bias the study of ICE, and therefore, was not included in this study. ICE elements identified in *R.anatipestifer* were named ICE*Ran* in accordance with the nomenclature system proposed by Burrus, the different ICE*Ran* elements were allocated to different strain numbers, ICE*Ran*RCAD0133-1, and ICE*Ran*RCAD0179-1 (23, 24). ICE*Ran*RCAD0133-1 was 70890 bp in size, carrying 77 protein coding sequences(CDSs) and inserted into the vicinity of hypothetical protein at position 991320-1062210, yielding 13 bp direct repeats (5'-AAGTTGAGTTACT-3') representing attL and attR sites. Similarly, ICE*Ran*RCAD0179-1 was a 49166 bp genomic region with 51 CDSs and inserted into position 54705-103871 of contig (QXQM00000004) of isolate RCAD0179 near an Arginyl-tRNA synthetase, where it yielded 7 bp direct repeats (5'-GTAACTT-3') located on either ends. The overall GC content of ICE*Ran* elements was 33.14% (ICE*Ran*RCAD0133-1) and 50.34% (ICE*Ran*RCAD0179-1). The G+C content of ICE*Ran*RCAD0133-1 (33.14%) was slightly lower than that of the rest of the chromosome (34.85% for the complete chromosomes of RCAD0133). However, the overall GC content of ICE*Ran*RCAD0179-1 was 50.34%, which was dramatically higher than the genomic GC content (34.58%) of *R. anatipestifer* RCAD0179. Comparison analysis of ICE*Ran*RCAD0133-1 and ICE*Ran*RCAD0179-1 was carried out through the BLASTn and BLASTp, which indicated that these two ICEs were not similar in DNA sequences or protein productions encoded by core genes and therefore were not classified into the same ICE family (11). Next, ICEberg server analysis was performed to exactly identify to which ICE family the ICE*Ran* elements belongs; consequently, ICE*Ran*RCAD0179-1 was classified into the CTnDOT/ERL family. Nevertheless, ICE*Ran*RCAD0133-1 was considered a novel ICE family because of low similarity to previously reported ICEs.

Active ICEs have the ability to excise from the host chromosome with the help of integrase and excisionase, and form an extrachromosomal circular form of the ICE (25). Therefore, to detect the circular intermediate, four pairs of primer pairs were designed that specifically targeted the DRs of ICERanRCAD0133-1 and ICERanRCAD017-1. Surprisingly, no expected product was observed (data not shown).

### Characterization of Cargo Genes Encoded by ICE*Ran* Elements

The cargo genes carried by ICEs often endow novel functions to the host bacteria affecting its life styles and niche adaption, such as development of virulence and acquired of antibiotic resistances (26).

For accessory gene of ICE*Ran*RCAD0133-1, it has a Toprim domain protein, which is a catalytic domain found in type IA and II topoisomerases, OLD family nucleases, and RecR proteins, proteins that are involved in DNA replication (27). This ICE had two genes that encode a bacteriocin/lantibiotic efflux ABC transporter. Bacteriocins are small ribosomally-synthesized peptides, that display a highly potent antimicrobial activity. The bacteriocin/lantibiotic efflux ABC transporter was composed of two functional domains: a peptidase C39 domain, which was involved in the processing of bacteriocin precursors at the double glycine, and an ABC transporter domain, which was involved in the export of bacteriocins (28, 29). An arylsulfatase regulator encoded by ICE*Ran*RCAD0133-1 can play a significant role in a response against sulfate-limiting conditions to fulfill its sulfate requirements in the bacterial cell (30–32). Interestingly, this ICE also had two genes encoding a putative TonB-dependent receptor, which was involved in siderophore-mediated iron acquisition and played a significant role in bacterial virulence of *R.anatipestifer* (33). ICE*Ran*RCAD0133-1 encoded a mycobacteriophage Barnyard protein gp56 (TIGR03299), which was found in different mycobacterium phage genomes, in *Streptomyces coelicolor* plasmid SCP1, and in bacterial genomes near various markers that suggest lateral gene transfer. Five insertion sequences (IS) were encoded by this ICE, which were all closely related to ISRa1, first found on the *R.anatipestifer* plasmid pCFC2, that belongs to the IS982 family; and is widely distributed in various *R.anatipestifer* strains with 2–20 copies (34).

A special region of isolate RCAD0179 was ICE*Ran*RCAD0179-1. Furthermore, comparison analysis revealed that a ribosomal protection type tetracycline resistance gene (*tetQ*) was carried by this ICE, which is an important contributor to the wide distribution of antibiotic resistance loci. A gene containing a lysozyme_like domain (cl00222) was found on ICE*Ran*RCAD0179-1, which is involved in the hydrolysis of β-1,4-linked polysaccharides. Bifunctional deaminase-reductase is involved in catalyzing the second and third steps in the biosynthesis of riboflavin. One such protein was present in ICE*Ran*RCAD0179-1, which contained a bifunctional deaminase-reductase domain (35). A gene encoding for prokaryotic ATPase was also identified in this ICE, and it may serve as a significant energy provider for the biological activity of ICE*Ran*RCAD0179-1 through ATP hydrolysis.

### Core Modules of ICE*Ran* Elements Involved in Integration and Excision, Regulation, and Conjugative Transfer

The two ICE*Ran* elements were not identical, and comparative analysis of the coding sequences (CDS) within the conserved region of ICE*Ran* elements confirmed the presence of core genes involved in ICE integration and excision, regulation, and maintenance, replication, and conjugative transfer.

In most cases, tyrosine integrase, a site-specific recombinase, frequently serves as a mediator of chromosomal integration and excision of ICEs, while in some cases, it can be replaced by a serine recombinase or DDE transposase as well. Two integrases were identified at one extremity of ICE*Ran*RCAD0133-1 and ICE*Ran*RCAD0179-1, respectively. An alignment of these two integrases with the characterized recombinases of the tyrosine integrase family suggested that they both contain a characteristic RHRH tetrad with conserved nucleophilic tyrosine at the C-terminus (5, 36). Therefore, they were grouped into the tyrosine integrase family (Supplementary Figure 2). A phylogenetic analysis was performed on the two integrases from ICE*Ran* elements with all available tyrosine integrases from the GenBank database. The phylogenetic tree showed that the integrase of ICE*Ran*RCAD0133-1 was phylogenetically distinct from other integrases and belonged to a single phylogenetic cluster but was close to *Ornithobacterium rhinotracheale* (Supplementary Figure 3). For another integrase of ICE*Ran*RCAD0179-1, it was closer to and grouped with integrases of the *Bacteroides* phylum (Supplementary Figure 4).

A set of various genes encoding for regulation and conjugative transfer was detected on ICE*Ran* elements; for ICE*Ran*RCAD0133-1, including three conjugative DNA processing and transfer clusters (*mobI, traABD, traEFGGIJKMNOQ*), regulators of excision and conjugative transfer (*AbiEi, TetR, and RteC*), A single-stranded DNA-binding protein and a DNA topoisomerase that are involved in DNA replication, recombination and repair, and often participate in single-stranded DNA recombination and replication intermediates, respectively, were also found in this ICE (5, 37). Furthermore, ICE*Ran*RCAD0133-1 also encoded a putative DNA methylase, which contains a conserved protein N6_Mtase (cl28090) domain, a member of restriction-modification (R-M) systems. These solitary methylases might provide a broad protection from the host R–M systems (38). Among the conserved core genes of ICE*Ran*RCAD0179-1, two clusters encoding for ICE conjugation processing and transfer (*mobI, mobII, traABCDEGHIJKMNPQ*) were identified. Moreover, this ICE had a series of two-component systems for ICE maintenance and regulation (*RteA, RteB, RteC*) (39, 40). A putative DNA methylase and a DNA topoisomerase III participated in ICE replication, recombination and repair (4). Therefore, all the core genes of the ICE*Ran* elements together ensured integration and excision, conjugative transfer, and regulation of ICE*Ran* elements.

### Homologous Regions of ICE*Ran* Elements in Related Species

**Figure 2:**
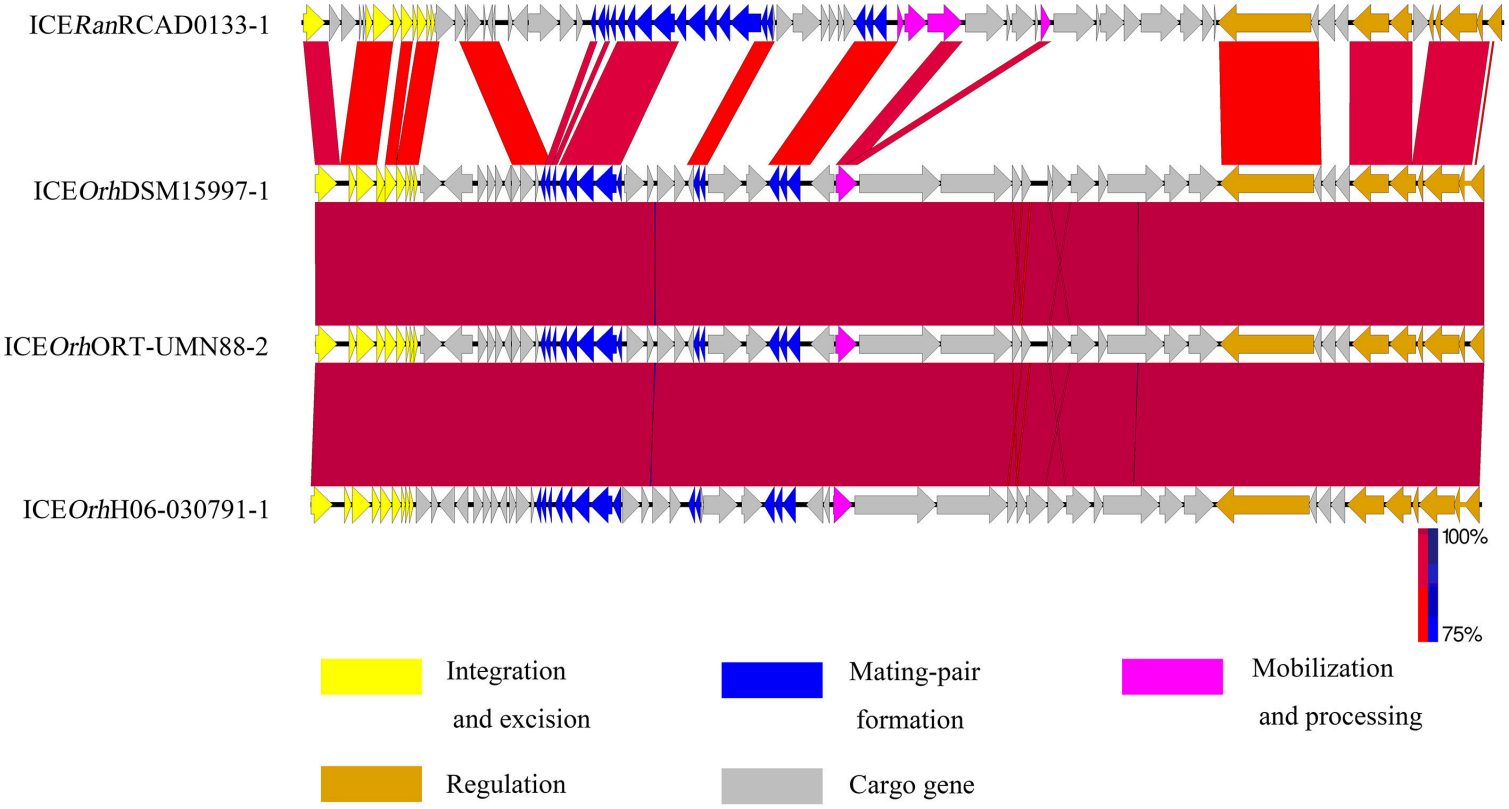
Comparison of the genetic organization of ICE*Ran*RCAD0133-1 with its closely related ICEs. Comparison of the genetic organization of ICE*Ran*RCAD0133-1 with its homologs in related bacteria. Its closely related ICEs were named with the nomenclature system list in Table S5. Arrows represent predicted CDSs. Highly conserved regions determined by pairwise BLASTn comparisons with E-values lower than 0.001 were plotted. Regions with forward and reverse matches are indicated by red and blue shades, respectively, with color intensity indicating nucleotide identity levels (from 75 to 100%). The absence of red and blue areas denotes no homology. ORFs with different functions are colored differently.

**Figure 3:**
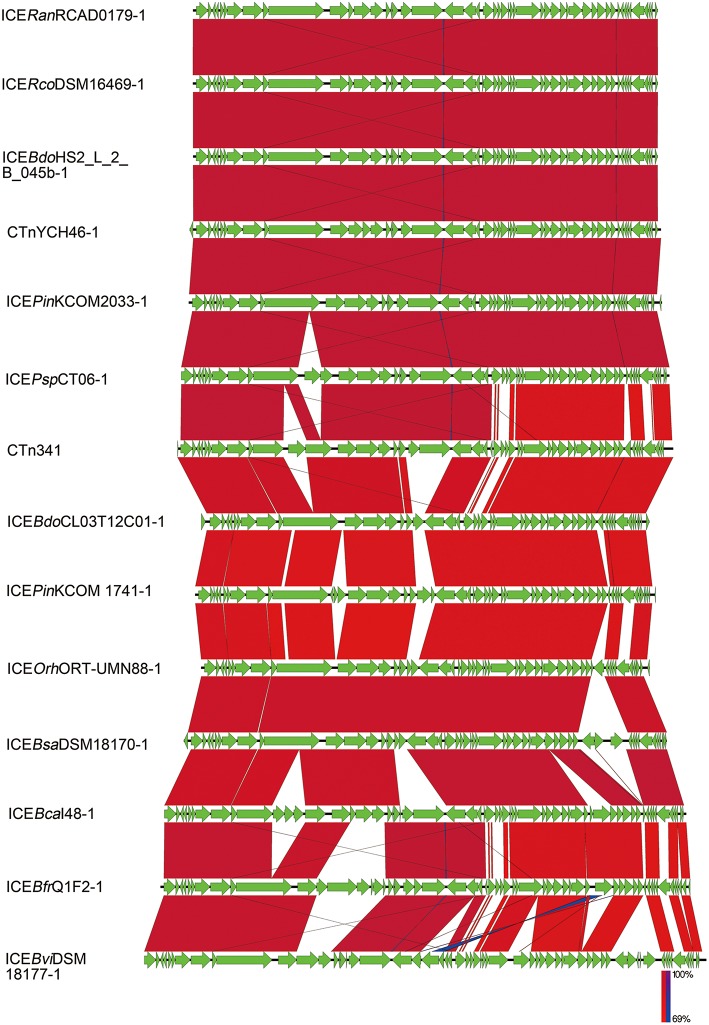
Comparison of the genetic organization of ICE*Ran*RCAD0179-1 with its closely related ICEs. Comparison of the genetic organization of ICE*Ran*RCAD0179-1 with its homologs in related bacteria. Its closely related ICEs were named with the nomenclature system list in Table S6. Arrows represent predicted CDSs. Highly conserved regions determined by pairwise BLASTn comparisons with E-values lower than 0.001 were plotted. Regions with forward and reverse matches are indicated by red and blue shades, respectively, with color intensity indicating nucleotide identity levels (from 69 to 100%). The absence of red and blue areas denotes no homology.

Nucleotide sequence alignments were performed to identify homologs of ICE*Ran* elements among the sequences of ICE-like elements available in the GenBank database, using the nucleotide sequence of ICE*Ran* elements as a probe. BLASTn analysis of the ICE in RCAD0133 against the NCBI nr database, suggested that three ICE-like elements similar to ICE*Ran*RCAD0133-1 were identified in *Ornithobacterium rhinotracheale* specie (Table S5). They shared the core backbone for integration, maintenance, and dissemination, and had significant similarity (BLASTp, coverage≥99.0%, identity≥81.0%, E-value 0.0). Comparison among integrases of ICE*Ran*RCAD0133-1 and the three ICE-like elements, confirmed that they belonged to the same ICE family (Figure 2). Interestingly, bioinformatics analysis for ICE*Ran*RCAD0179-1 suggested that 13 ICE-like elements resemble ICE*Ran*RCAD0179-1 and are widely distributed in the genome sequences of several different bacteria including(Table S6):*Prevotella intermedia* strain KCOM 2033, *Bacteroides dorei* isolate HS2_L_2_B_045b, *Bacteroides fragilis* YCH46 DNA, *Parabacteroides sp*. CT06, *Bacteroides caecimuris* strain I48, *Barnesiella viscericola* DSM 18177, *Ornithobacterium rhinotracheale* ORT-UMN 88, *Bacteroides salanitronis* DSM 18170,*Bacteroides fragilis* strain Q1F2,*Bacteroides dorei* CL03T12C01,*Prevotella intermedia strain* KCOM 1741,and *Riemerella columbina* DSM 16469. Based on sequence similarity and structural comparison with the backbone of core genes in ICE*Ran*RCAD0179-1, a member of the CTnDOT/ERL family, these putative ICE elements were also classified into the CTnDOT/ERL family. Comparison between ICE*Ran* elements and their closely related ICE-like elements was performed with the genome comparison visualizer Easyfig (Figure 3).

### Phylogenetic Relationship Between ICE*Ran*RCAD0179-1 and Related ICE-like Elements

**Figure 4:**
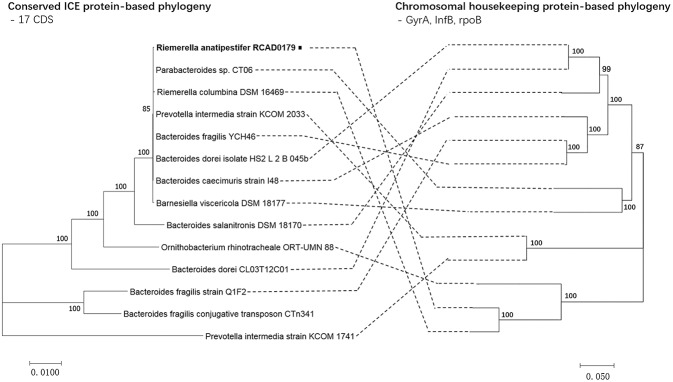
Phylogeny of ICE*Ran*RCAD0179-1 and closely related ICEs and comparison to chromosomal housekeeping marker phylogeny. Alignments were performed with MUSCLE using the concatenated amino acid sequences of the ICE elements (17 conserved CDSs) and housekeeping markers (gyrA, infB, rpoB). Phylogenies were constructed using MEGA v 7.0, using the neighbor-joining algorithm, Poisson correction and bootstrap analysis (*n* = 1000). In genbank, CTn341 only had its own sequence, and its host bacterial sequence wasn't in publicly available.

**Figure 5:**
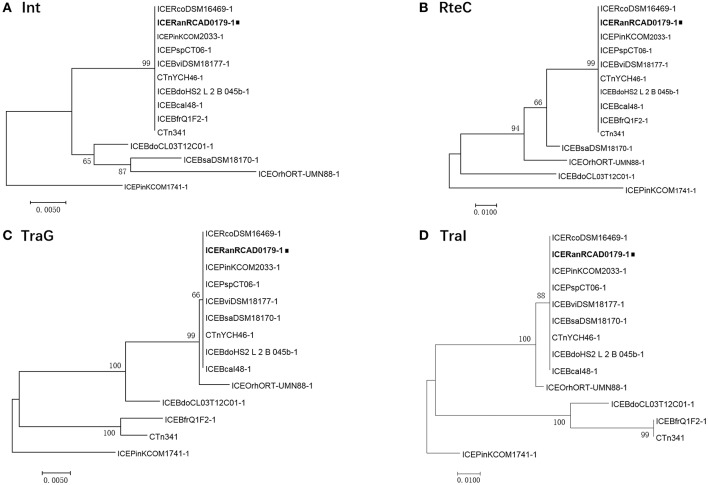
Phylogenetic analysis of ICE*Ran*RCAD0179-1 **(A)** Int, **(B)** RteC, **(C)** TraG, and **(D)** TraI. The trees were constructed by applying the maximum likelihood method based on the Poisson correction model using MEGA v 7.0. Bootstrap analysis with 1,000 replications was performed to test the reliability of each tree.

A high level of synteny was also indicated by alignments of ICE*Ran*RCAD0179-1 and 13 closely related ICEs. The ICE phylogenetic tree was constructed based on the concatenated amino acid sequences of 17 conserved proteins (*mob1, mob2, RteC, Top, TraD, TraE, TraF, TraG, TraH, TraI, TraJ, TraK, TraL, TraM, TraO, TraP, TraQ*) encoded by ICE*Ran*RCAD0179-1 and the closely related ICE-like elements to speculate on the evolution of these ICEs. Comparison of the phylogenetic tree topologies produced by neighbor-joining analysis of concatenated amino acid sequences of the conserved ICE CDS and protein products of ICE hosts chromosomal housekeeping gene (*gyrA, infB, rpoB*) showed that ICE*Ran*RCAD0179-1 was clustered with most ICE-like elements in *Bacteroides*, and the ICE-like element in *Riemerella columbina* DSM 16469, and the evolutionary relationship of ICEs wasn't consistent with that of the ICE host genes (Figure 4). Furthermore, to trace the evolutionary relationship among these ICEs and anticipate their ancestral root, a phylogenetic tree was produced based on four proteins (*Int, TraG, TraI, RteC*) corresponding to significant ICE function, such as integration, exclusion determination, transfer, and regulation, respectively. Our analysis showed that ICE*Ran*RCAD0179-1 and homologous ICE-like elements in *Bacteroides* were very closely related and clustered into one branch, while the ICE*Pink*COM1741-1 always formed a separate branch (Figure 5).

## Discussion

Genomic sequence analysis first revealed two ICEs (ICE*Ran*RCAD0133-1 and ICE*Ran*RCAD0179-1) in the *R.anatipestifer* genomes of RCAD0133 and RCAD0179, respectively. The ICE family is generally determined by the definition that they should have an integrase homology of ≥60% and significant structural synteny. Based on the significant similarity of ICE core modules, ICE*Ran*RCAD0133-1 and its related ICEs were grouped in one novel ICE family, due to lack of similarities to known ICE families (11). Similarly, ICE*Ran*RCAD0179-1 and its 13 related ICEs were classified into the same ICE family (CTnDOT/ERL). The high prevalence of ICE-like elements similar to ICE*Ran*RCAD0179-1 indicated that interspecies genetic exchange also occurred via ICEs, thus broadening the host range, and dissemination of combined cargo genes across different strains. In accordance with the phylogenetic tree of the integrases of these two ICEs, the integrase of ICE*Ran*RCAD0133-1 was grouped in a cluster with *Ornithobacterium rhinotracheale* specie, and the integrase of ICE*Ran*RCAD0179-1 had a close phylogeny relationship with *Bacteroides* phylum because they were in one clade of the phylogenetic tree. Interestingly, integrases of the two ICEs were both classified in the tyrosine integrase family. Therefore, it can be envisaged that the two ICEs may have a similar integration or excision behavior for mobile element DNA because integrases of the ICE*Ran* elements are in the same family.

ICEs are self-transmissible mobile genetic elements that have core modules for conjugative transfer and intricate regulatory systems to control their excision from the chromosome (1). Although integrative and conjugative genes were present in ICE*Ran*RCAD0133-1 and ICE*Ran*RCAD0179, to date we failed to detect a circular intermediate by PCR method via repeated experiments, which suggests that these two ICE elements may have lost the ability to recircularize and transfer to other bacteria.

ICE*Ran*RCAD0133-1 had a similar GC content to the host genome, and it can be envisaged that this ICE has been assimilated by the host genome at an early evolutionary timepoint or comes from the bacteria with similar GC content to RCAD0133. Cargo genes encoded on this ICE greatly expanded *R.anatipestifer* genome variability. R-M systems usually protect a bacterial cell against invasion of foreign DNA by endonucleolytic cleavage of DNA that is unmethylated, and also provide resistance against bacteriophages. The solitary methylase encoded on this ICE might provide a broad protection from the host R–M systems (41). IS elements often encode transposase(s) which can insert randomly into various DNA sites, utilize the replicative mechanisms to generate a copy at a new position, are frequently implicated in ICE recombination, and cause genomic rearrangements including deletion, inversion and cointegrate formation (42). Therefore, ISs shape the host bacterial genome and facilitate the diversity of microbes. Arylsulfatase has been implicated in *E. coli* infection of the brain microvascular endothelial cells (BMEC) of the host. The presence of arylsulfatase may contribute to the ability of the pathogen to cross the blood-brain barrier leading to meningitis (30–32). Interestingly, an arylsulfatase regulator was present on this ICE, and we hypothesize that it may be implicated in the pathogenicity of *R.anatipestifer* through participation in the regulation process of arylsulfatase. Based on previous research, we conferred that the putative TonB-dependent receptor encoded on this ICE may significantly influence *R.anatipestifer* pathogenicity via hemin and iron acquisition. Bacteriocins are synthesized ribosomally, and always display highly potent antimicrobial activity, their production relies on genes encoding a bacteriocin, a bacteriocin ABC transporter, and bacteriocin immunity proteins (43). The bacteriocin/lantibiotic efflux ABC transporter detected on this ICE may participate in the export of peptide bacteriocins across the cytoplasmic membrane. The cargo genes carried on this ICE, with potential for pathogenesis along with other genes in the RCAD0133 strain, also expanded bacterial genome diversity, which may provide further insights into the adaptation mechanisms of the pathogen to thrive under diverse ecological niches.

The ICE element identified in isolate RCAD0179 had a significantly higher GC content than the host genome, which implied that this region was not native and had been adopted by HGT. Comparative analysis confirmed that it belonged to the CTnDOT/ERL family. The spread of resistance determinants was mostly contributed by the conjugative transfer of the CTnDOT/ERL element within the *Bacteroides* group (44). A tetracycline resistance gene present on ICE*Ran*RCAD0179-1 will be an important vehicle for the wide distribution of antibiotic resistance loci. It provided a competitive advantage for the host to rapidly overcome harsh environment. Comparison of the phylogenetic trees of ICE*Ran*RCAD0179-1 and related ICE elements against their hosts demonstrated that the compared ICEs were derived from a common ancestor that underwent divergence prior to integration into the host bacterial chromosome. Furthermore, through analysis of the phylogenetic trees based on the single core gene of these ICEs, it was identified that they shared almost the same topology structure of phylogeny, which indicated that the individual core genes most likely have not evolved independently; rather; these ICEs underwent a related evolutionary process or experienced only a low degree of recombination events during spread from a common CTnDOT/ERL family ancestor.

In conclusion, genomic analysis revealed two ICE*Ran* elements in *R.anatipestifer* for the first time. *In silico* characterization of the ICE*Ran*RCAD0179-1 elements showed that similar ICE-like elements were found in other *Bacteroides* and that these ICEs are likely derived from a common ancestor. The two ICE*Ran* elements possess the core elements required for ICE DNA integration, maintenance and regulation, and dissemination. The presence of these core modules, however, can't prove that the ICE*Ran* elements can excise, circularize, conjugative transfer and then integrate within the chromosome. Genes encoding resistance to tetracycline antibiotics, DNA repair and recombination systems, virulence factors, toxin-antitoxin system, and regulation of conjugative transfer, were found to be present within cargo genes of ICE*Ran* elements. In particular, the putative antibiotic biosynthetic loci encoded on ICE*Ran* elements provide potential targets for the exploration of *R.anatipestifer* antibiosis phenotypes. ICE*Ran* elements could therefore play a significant role in providing beneficial phenotypes to their hosts. With the emergence of massive amounts of available genome sequences, it can be reasonably envisaged that more novel ICE elements will be identified and novel ICE families will be described. It may assist in studying the special mechanisms by which ICE impacts host metabolism and physiology.

## Author Contributions

DZ and AC conceived and designed the project. JW, JX, LZ, YL, and XC cultured bacteria and extracted genomic DNA. ZY, ML, RJ, SC, JX, and MW assembled and annotated the genomes. JW, XZ, QY, YY, and YW performed genomic islands analysis. JW, ZY, and SZ performed ICE identification and related ICE search. JW, DZ, and ZY performed the construction of the phylogenetic tree. JW and DZ drafted and revised the manuscript. All authors have read and approved the final version manuscript.

### Conflict of Interest Statement

The authors declare that the research was conducted in the absence of any commercial or financial relationships that could be construed as a potential conflict of interest.
