# Supplementary material for: First Report of Integrative Conjugative Elements in Riemerella anatipestifer Isolates From Ducks in China
Source: Front Vet Sci. 2019 Apr 24;6:128. doi: 10.3389/fvets.2019.00128 (PMC6491836; doi:10.3389/fvets.2019.00128)
Supplement: Supplementary file 1 [file Table_1.pdf]

Additional file 1 Table S1 The genomic status and accession number of 48 *R. anatipestifer* genomes

name	Isolated location	Isolated date	Host	size(Mb)	GC%	Accession number	Gene	Protein	Complete(C)/ Draft(No. of contigs)
ATCC 11845	USA	1932	Duck	2.16409	35	CP003388.1	2062	1993	C
RA-GD	China	2011	Duck	2.16638	35	CP002562.1	2063	1895	C
RA-CH-1	China	1993	Duck	2.30952	35.1	CP003787.1	2207	2104	C
RA-CH-2	China	1996	Duck	2.16632	35	CP004020.1	2050	1969	C
CH3	China	2013	Duck	2.23448	35.2	CP006649.1	2119	1971	C
strain 153	China	2014	Duck	2.16028	35	CP007504.1	2045	1801	C
strain 17	China	2014	Duck	2.15252	35	CP007503.1	2080	1810	C
Yb2	China	2014	Duck	2.18407	35	CP007204.1	2091	2021	C
HXb2	China	2015	Duck	2.42524	35	CP011859.1	2367	2238	C
RA-SG	China	2012	Duck	2.17263	35	ANGF00000000	2081	1985	31
RA-YM	China	2010	Duck	2.1302	35	AENH01000000	2030	1959	29
RA-JLLY	China	2015	Duck	2.18797	35.1	LAVB01000000	2063	1970	16
RCAD0122	China	2012	Duck	2.19865	35	LUDU01000000	2095	2028	37
RCAD0142	China	2011	Duck	2.10327	35	LUDG01000000	1967	1906	17
RCAD0188	China	2014	Duck	2.15653	35	LUDH01000000	2022	1956	61
RCAD0125	China	2012	Duck	2.13824	35	LUDJ01000000	2010	1934	30
RCAD0150	China	2011	Duck	2.09889	34.9	LUDM01000000	1963	1901	28
RCAD0123	China	2011	Duck	2.08621	35	LUDP01000000	1955	1891	26
RCAD0124	China	2011	Duck	2.19889	35	LUDQ01000000	2097	2028	50
RCAD0133	China	2011	Duck	2509140	34.8	CP029760	2471	2273	C
RCAD0183	China	2014	Duck	2.20403	35	LUDK01000000	2090	2019	52
RCAD0147	China	2011	Duck	2.0982	35	LUDN01000000	1967	1901	36
RCAD0134	China	2011	Duck	2.153	35	LUDO01000000	2029	1961	40
RCAD0131	China	2011	Duck	2.10396	35	LUDS01000000	1974	1910	24
RCAD0127	China	2013	Duck	2.08862	35	LUDV01000000	1955	1891	21
RCAD0121	China	2011	Duck	2.2167	35.2	LUDI01000000	2090	1989	22
RA1	India	2016	Duck	2.15701	35	PPEC01000000	2047	1959	56
17CS0503	Germany	2018	Duck	2.12739	35	PKKR01000000	2011	1943	31
RA2	India	2018	Duck	1.99293	35	QEWX00000000	2037	1711	137
RCAD0181	China	2014	Duck	2.11542	35.1	LUDL01000000	1981	1911	71
RCAD0111	China	2012	Duck	2.35718	35.1	LUDR01000000	2270	2144	63

RCAD0135	China	2017	Duck	2286048	35	QXQO00000000	2,199	2,096	32
RCAD0152-1	China	2011	Duck	2236324	35.1	QXQN00000000	2,142	2,040	28
RCAD0179	China	2013	Duck	2205109	35.2	QXQM00000000	2,126	2,048	37
RCAD0282	China	2014	Duck	2285175	35	QXQL00000000	2,202	2,098	34
RCAD0377	China	2017	Duck	2094374	34.9	QXQK00000000	1,983	1,912	36
RCAD0421	China	2017	Duck	2120678	35	QXQJ00000000	1,986	1,887	34
RCAD0422	China	2017	Duck	2119439	35	QXQI00000000	1,990	1,890	36
RCAD0427	China	2017	Duck	2261483	35	QXQH00000000	2,163	2,057	33
CCUG18373	USA	1955	Duck	2149492	34.9	QXHN00000000	2057	1986	25
CCUG25001	UK	1966	Duck	2299422	34.8	QXHO00000000	2254	2108	70
CCUG25002	UK	1976	Duck	2332779	34.8	QXHP00000000	2497	2293	257
CCUG25004	UK	1976	Duck	2139715	34.9	QXHQ00000000	2043	1971	34
CCUG25005	UK	1966	Duck	2167466	34.8	QXHR00000000	2197	2024	163
CCUG25008	UK	1966	Duck	2291094	34.8	QXHS00000000	2248	2105	77
CCUG25010	UK	1976	Duck	2414224	34.8	QXHT00000000	2601	2401	287
CCUG25054	UK	1976	Duck	2308571	35.2	QXHU00000000	2246	2106	57
CCUG25055	USA	1976	Duck	2265911	34.7	QXHV00000000	2195	2061	57
