# Supplementary material for: First Report of Integrative Conjugative Elements in Riemerella anatipestifer Isolates From Ducks in China
Source: Front Vet Sci. 2019 Apr 24;6:128. doi: 10.3389/fvets.2019.00128 (PMC6491836; doi:10.3389/fvets.2019.00128)
Supplement: Supplementary file 5 [file Table_5.pdf]

Additional file 5 Table S5 The genomic status of ICE*Ran*RCAD0133-1 and related ICE

Host Name	GC%(host)	Accession number	ICE Name	GC%(ICE)	ICE length	ICE location	ICE coverage	ICE identity
Riemerella anatipestifer RCAD0133	34.8	CP029760	ICE*Ran*RCAD0133-1	33.1	70890 bp	991320..1062210		
Ornithobacterium rhinotracheale ORT-UMN 88	37.4	CP006828.1	ICE*Orh*ORT-UMN88-2	34.6	67121 bp	1993107..2060227	43%	90%
Ornithobacterium rhinotracheale DSM 15997	37.2	CP003283.1	ICE*Orh*DSM15997-1	34.6	67131 bp	2041421..2108551	43%	90%
Ornithobacterium rhinotracheale H06-030791	37.2	AXDE01000007.1	ICE*Orh*H06-030791-1	34.5	67631 bp	372225..439855	43%	90%
