# Supplementary material for: First Report of Integrative Conjugative Elements in Riemerella anatipestifer Isolates From Ducks in China
Source: Front Vet Sci. 2019 Apr 24;6:128. doi: 10.3389/fvets.2019.00128 (PMC6491836; doi:10.3389/fvets.2019.00128)
Supplement: Supplementary file 6 [file Table_6.pdf]

**Additional file 6 Table S6 The genomic status of ICE*Ran*RCAD0179-1 and related ICE**

| Host Name | GC%(host) | Accession number | ICE Name | GC%(ICE) | ICE length | ICE location | ICE coverage | ICE identity |
|---|---|---|---|---|---|---|---|---|
| *Riemerella anatipestifer* RCAD0179 | 34.5 | QXQM00000004 | ICE*Ran*RCAD0179-1 | 50.3 | 49166 bp | 54705-103871 | | |
| *Prevotella intermedia* strain KCOM 2033 | 43.7 | CP024696.1 | ICE*Pin*KCOM2033-1 | 50.1 | 49621 bp | 2702780-2752400 | 93% | 99% |
| *Bacteroides dorei* isolate HS2_L_2_B_045b | 42 | CP009057.1 | ICE*Bdo*HS2_L_2_B_045b-1 | 50.3 | 48721 bp | 1637634-1686354 | 93% | 99% |
| *Bacteroides fragilis* YCH46 | 43.2 | AP006841.1 | CTnYCH46-1(Kuwahara et al., 2004) | 50.1 | 49428 bp | 114772-164199 | 93% | 99% |
| *Parabacteroides sp*. CT06 | 45.1 | CP022754.1 | ICE*Psp*CT06-1 | 50.1 | 51188 bp | 512165-563352 | 93% | 99% |
| *Bacteroides caecimuris* strain I48 | 42.6 | CP015401.2 | ICE*Bca*I48-1 | 49.5 | 54718 bp | 1448812-1503529 | 93% | 99% |
| *Barnesiella viscericola* DSM 18177 | 51.6 | CP007034.1 | ICE*Bvi*DSM18177-1 | 48.9 | 58956 bp | 2851185-2910140 | 93% | 99% |
| *Bacteroides fragilis* | | AY515263.1 | CTn341(Bacic et al., 2005) | 49.4 | 51993 bp | 1-51993 | 86% | 99% |
| *Ornithobacterium rhinotracheale* ORT-UMN 88 | 37.4 | CP006828.1 | ICE*Orh*ORT-UMN88-1(Zehr et al., 2014) | 50.3 | 47024 bp | 2117077-2164100 | 83% | 95% |
| *Bacteroides salanitronis* DSM 18170 | 46.6 | CP002530.1 | ICE*Bsa*DSM18170-1 | 50.2 | 50650 bp | 2939351-2990000 | 84% | 99% |
| *Bacteroides fragilis* strain Q1F2 | 43.5 | CP018937.1 | ICE*Bfr*Q1F2-1 | 49.7 | 55555 bp | 2953654-3009208 | 90% | 97% |
| *Bacteroides dorei* CL03T12C01 | 41.5 | CP011531.1 | ICE*Bdo*CL03T12C01-1 | 51.2 | 46989 bp | 2319000-2365988 | 81% | 91% |
| *Prevotella intermedia* strain KCOM 1741 | 44.9 | CP024733.1 | ICE*Pin*KCOM1741-1 | 51.7 | 48295 bp | 592106-640400 | 79% | 86% |
| *Riemerella columbina* DSM 16469 | 36 | ARFT01000009.1 | ICE*Rco*DSM16469-1 | 50.3 | 48754 bp | 30256-79009 | 93% | 99% |

Note: The names of ICE (integrative conjugative element) and ICE-like elements identified in this study which have not been described in Published literature are designated with a name according to classical nomenclature for transposable genetic elements (Burrus et al., 2002; Roberts et al., 2008).
