# Supplementary material for: First Report of Integrative Conjugative Elements in Riemerella anatipestifer Isolates From Ducks in China
Source: Front Vet Sci. 2019 Apr 24;6:128. doi: 10.3389/fvets.2019.00128 (PMC6491836; doi:10.3389/fvets.2019.00128)
Supplement: Supplementary file 7 [file Presentation_1.pdf]

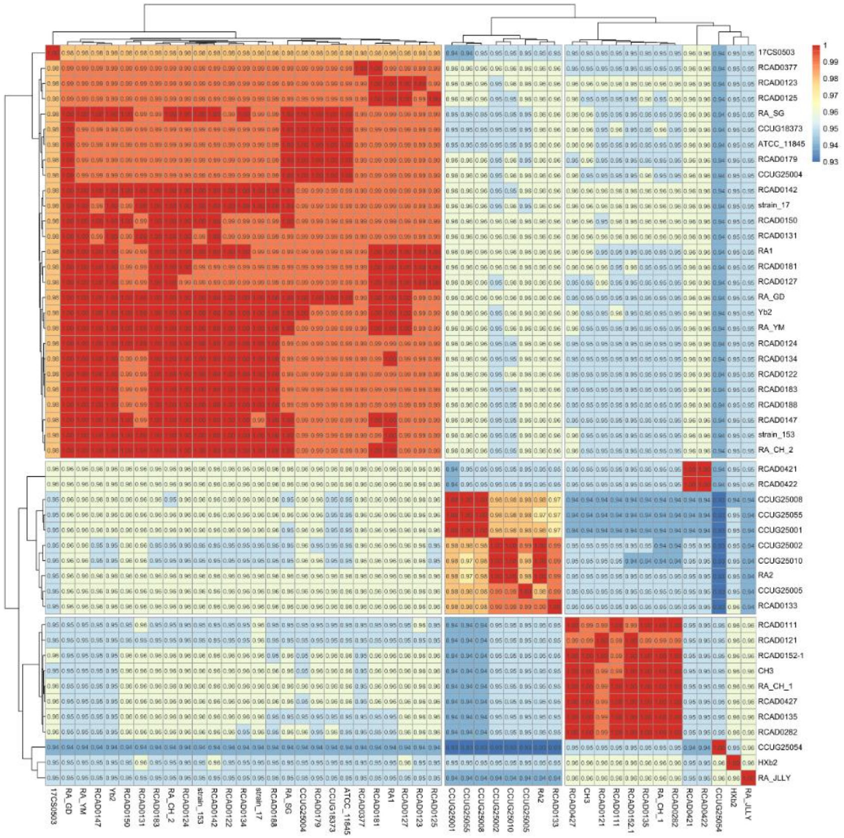

Supplementary Figure 1. Genome-based assessment of species of *R.anatipestifer*. Heat map of Average Nucleotide Identity (ANI) values of 48 *R.anatipestifer* isolates.

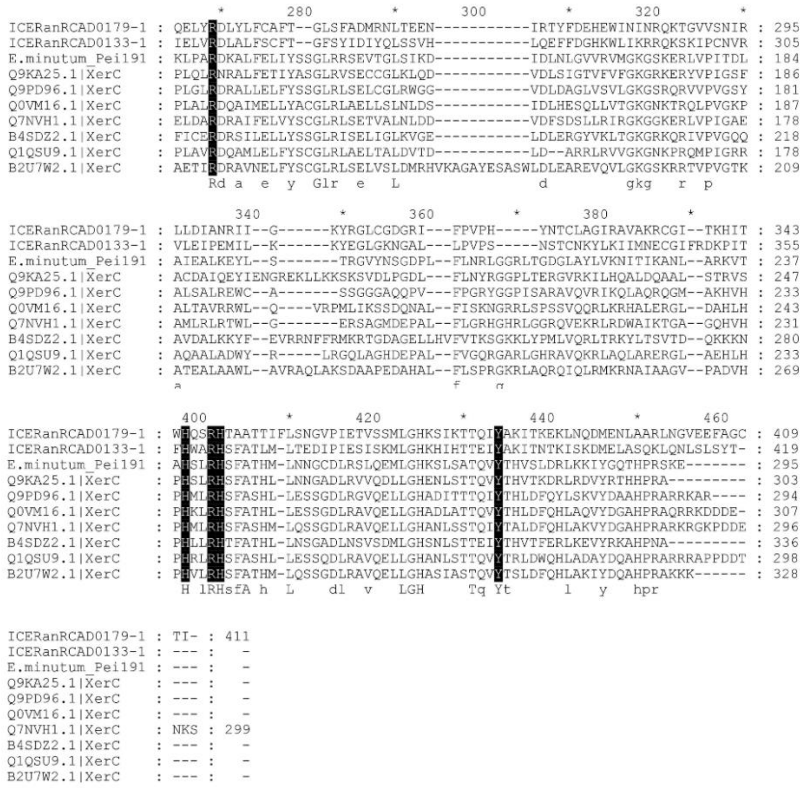

Supplementary Figure 2. Analysis of integrase of ICE*Ran* elements. Alignment of the conserved domains of recombinases of ICE*Ran* elements with the characterized recombinases of the tyrosine integrase family revealing conserved RHRH tetrad and conserved tyrosine which is a distinguishing feature of tyrosine recombinase superfamily.

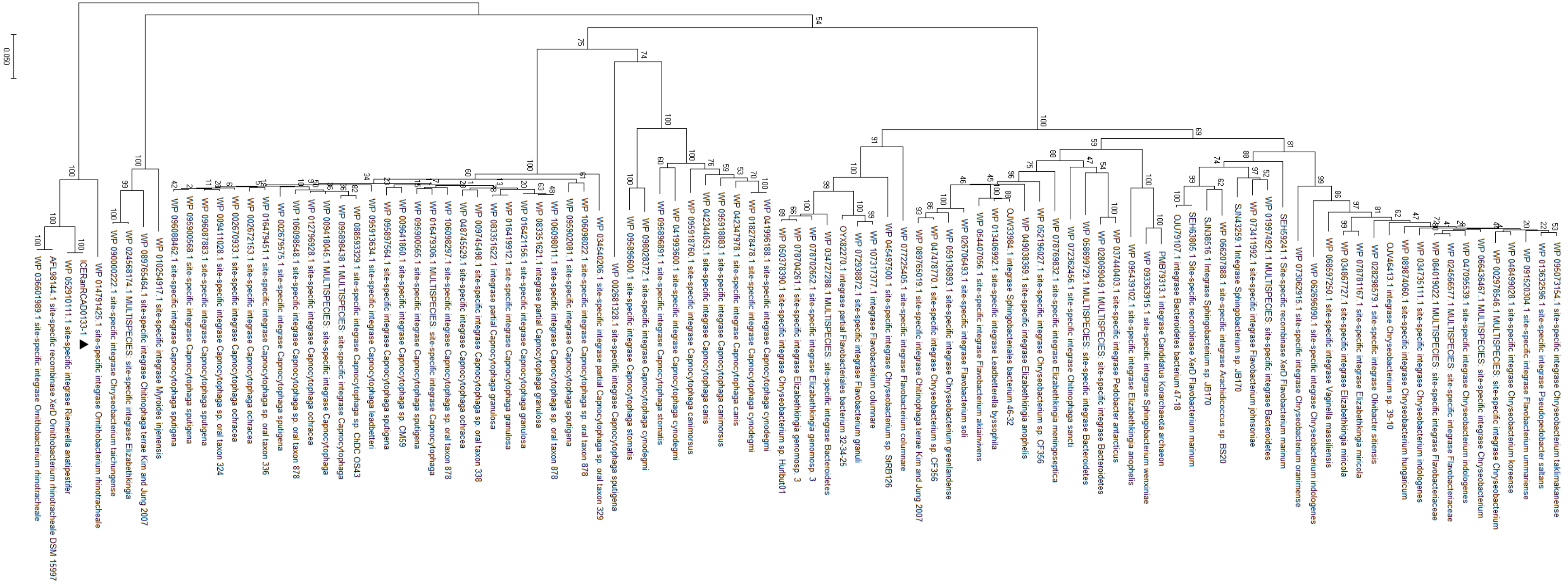

Supplementary Figure 3. Phylogenetic tree of tyrosine recombinase like protein from phages, prophages and ICE available at GenBank database with recombinase of ICE*Ran*RCAD0133-1 (highlighted). The scale bar represents a 0.05 substitution per nucleotide position.

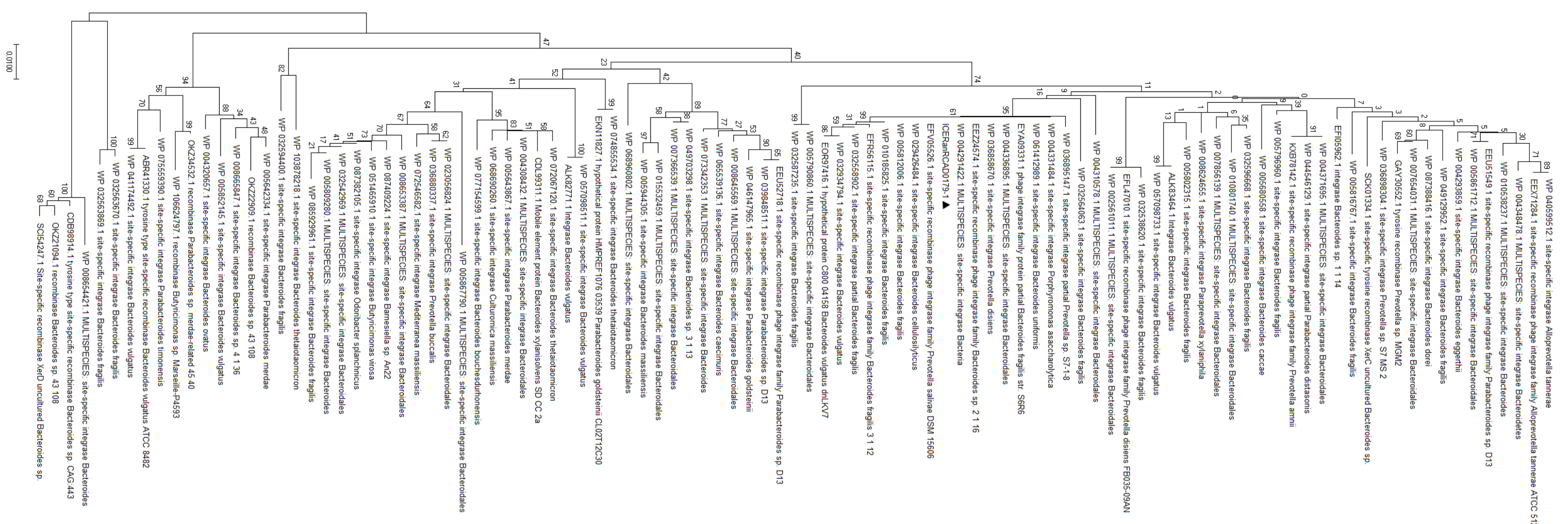

Supplementary Figure 4. Phylogenetic tree of tyrosine recombinase like protein from phages, prophages and ICE available at GenBank database with recombinase of ICE*Ran*RCAD0179-1 (highlighted). The scale bar represents a 0.01 substitution per nucleotide position.

Reference:

Bacic, M., Parker, A.C., Stagg, J., Whitley, H.P., Wells, W.G., Jacob, L.A., et al. (2005). Genetic and Structural Analysis of the Bacteroides Conjugative Transposon CTn341. *Journal of Bacteriology* 187(8)**,** 2858-2869. doi: 10.1128/jb.187.8.2858-2869.2005.

Burrus, V., Pavlovic, G., Decaris, B., and Guédon, G. (2002). The ICESt1 element of *Streptococcus thermophilus* belongs to a large family of integrative and conjugative elements that exchange modules and change their specificity of integration. *Plasmid* 48(2)**,** 77-97.

Kuwahara, T., Yamashita, A., Hirakawa, H., Nakayama, H., Toh, H., Okada, N., et al. (2004). Genomic analysis of *Bacteroides fragilis* reveals extensive DNA inversions regulating cell surface adaptation. *Proceedings of the National Academy of Sciences of the United States of America* 101(41)**,** 14919-14924. doi: 10.1073/pnas.0404172101.

Roberts, A.P., Chandler, M., Courvalin, P., Guédon, G., Mullany, P., Pembroke, T., et al. (2008). Revised nomenclature for transposable genetic elements. *Plasmid* 60(3)**,** 167-173.

Zehr, E.S., Bayles, D.O., Boatwright, W.D., Tabatabai, L.B., and Register, K.B. (2014). Complete genome sequence of *Ornithobacterium rhinotracheale* strain ORT-UMN 88. *Standards in genomic sciences* 9(1)**,** 16.
